# Genome-wide association study and fine-mapping identify a major quantitative trait locus controlling hundred-seed weight in soybean

**DOI:** 10.3389/fpls.2025.1716186

**Published:** 2025-11-28

**Authors:** Chunlei Zhang, Huilong Hong, Rongqiang Yuan, Shiyao Zhang, Tianjiao Gao, Shuping Yan, Sobhi F. Lamlom, Honglei Ren, Zhangxiong Liu, Jiajun Wang

**Affiliations:** 1 Soybean Research Institute of Heilongjiang Academy of Agriculture Sciences, Harbin, China; 2 National Key Facility for Crop Gene Resources and Genetic Improvement, Institute of Crop Sciences, Chinese Academy of Agricultural Sciences, Beijing, China; 3 Plant Production Department, Faculty of Agriculture Saba Basha, Alexandria University, Alexandria, Egypt

**Keywords:** soybean, hundred-seed weight, genome-wide association study, quantitative trait loci, fine-mapping, marker-assisted selection, seed development

## Abstract

**Background:**

Hundred-seed weight (HSW) is a critical yield component in soybean that directly influences productivity and seed quality. Despite its agronomic importance, the genetic architecture underlying natural variation in seed weight remains incompletely understood.

**Methods:**

We conducted a comprehensive genome-wide association study (GWAS) using 554 globally diverse soybean accessions, comprising 453 Chinese varieties (81.8%) and 101 international accessions (18.2%) from 15 countries. Accessions were evaluated across three consecutive years (2022-2024) and genotyped with 78,050 high-quality single-nucleotide polymorphisms (SNPs).

**Results:**

Mixed linear model (MLM) analysis revealed a major QTL on Chr.20 that consistently explained the largest proportion of phenotypic variation across all environments. This QTL demonstrated exceptional temporal stability, maintaining genome-wide significance with peak -log_10_(P) values of 13.4, 12.1, and 10.2 across the three evaluation years. Fine mapping narrowed the critical interval to 493.69 kb containing 25 annotated genes. The lead SNP within *Glyma.20G223200* explained 8-12% of phenotypic variance, while multi-SNP models incorporating five high-priority candidates cumulatively explained 14-18% of variance. Expression analysis of candidate genes revealed differential patterns between large-seeded and small-seeded varieties during seed development, with up to 32-fold expression differences.

**Conclusions:**

The environmentally stable Chr. 20 QTL provides immediate opportunities for marker-assisted selection (MAS) in soybean breeding programs. Genomic prediction modeling suggests 35% greater genetic gain compared to phenotypic selection alone, supporting broad applicability for global soybean improvement programs.

## 1 Introduction

Soybean [*Glycine max* (L.) Merr.] serves as a cornerstone of global agriculture, providing approximately 70% of the world’s protein meal and 29% of vegetable oil production (Mishra et al., 2024). With global soybean demand projected to increase by 80% by 2050 due to population growth and rising protein consumption, enhancing yield potential remains critical for sustainable agricultural intensification (Banoth et al., 2025). Among the primary yield components plants per unit area, pods per plant, seeds per pod, and individual seed weight- HSW represents a fundamental trait that significantly impacts both productivity and economic returns (Soni, 2021).

The HSW exhibits substantial natural variation, ranging from less than 5 g in wild soybean (*Glycine soja*) to over 40 g in large-seeded cultivars, indicating considerable potential for genetic improvement (Elattar et al., 2021; Zhang et al., 2024). Unlike other yield components that environmental factors can highly influence, seed weight demonstrates relatively high heritability (h² = 0.60–0.85), making it an attractive target for MAS and genomic breeding approaches (Crosta, 2024). This stability across environments, combined with its direct impact on yield, positions HSW as a key trait for breeding programs worldwide (Bartaula, 2022). Traditional QTL mapping has identified numerous genomic regions associated with seed weight in soybean (Luo et al., 2023; Xu et al., 2023). However, these biparental population-based studies have been constrained by limited genetic diversity and relatively low mapping resolution, typically identifying confidence intervals spanning several megabases (Kumar et al., 2023). Most investigations have focused on specific geographic regions or breeding programs, potentially overlooking globally distributed genetic variation that could enhance breeding efficiency and expand the genetic base of commercial varieties (Liu et al., 2023; Li et al., 2025).

Genome-wide association studies (GWAS) offer significant advantages over traditional QTL mapping for dissecting complex quantitative traits (Li and Ritchie, 2021; Uffelmann et al., 2021). In soybean, GWAS has successfully identified loci associated with flowering time, plant height, disease resistance, and seed composition traits (Ravelombola et al., 2021; Shao et al., 2022). However, most previous GWAS of seed weight have utilized regionally focused collections of 200–500 accessions, primarily from North American or Asian breeding programs (Cao et al., 2022; Fortune, 2024). While providing valuable insights, these studies may have limited power to detect rare variants or those specific to geographic regions, and the relatively modest population sizes may have constrained the identification of small-effect loci that collectively contribute to trait variation.

Recent advances in high-throughput genotyping technologies have enabled cost-effective genome-wide analysis of extensive germplasm collections (Guo et al., 2021). High-density SNP arrays provide sufficient marker coverage for effective GWAS while remaining economically feasible for large-scale studies (Altaf et al., 2024; Anokye et al., 2025). Concurrent improvements in statistical methodologies for handling population structure, kinship relationships, and multiple testing corrections have enhanced the reliability and interpretability of GWAS results (Saini et al., 2022). These technological and analytical advances create opportunities for conducting a more comprehensive genetic dissection of seed weight using globally diverse germplasm collections (Wang, 2024). The identification of genetic variants associated with seed weight has immediate practical applications for soybean breeding (Xue and Cui, 2025). Molecular markers linked to favorable alleles can accelerate variety development through marker-assisted selection, while reducing the requirements for phenotypic evaluation (Singh et al., 2022). Understanding the genetic architecture of seed weight can inform breeding strategies, including the optimal balance between selecting for major-effect loci versus polygenic approaches, and guide crossing program design to maximize genetic gain (Kumar et al., 2023).

This study addresses critical knowledge gaps by conducting comprehensive GWAS analysis using 554 globally diverse soybean accessions representing 16 countries. The collection comprises 453 accessions from China, providing an extensive sampling from the primary center of soybean diversity, complemented by 101 international accessions, which ensure broad representation of global genetic variation. The specific objectives were to characterize phenotypic and genetic diversity for HSW in a globally representative collection; identify genetic loci significantly associated with seed weight through genome-wide association analysis; fine-map major-effect loci to facilitate gene identification and functional characterization; and provide molecular tools and genetic resources for seed weight improvement in breeding programs worldwide. These findings will contribute to more efficient breeding strategies for yield improvement, while expanding our fundamental understanding of the genetic mechanisms controlling seed development in legume crops.

## 2 Materials and methods

### 2.1 Plant materials

**Figure 1:**
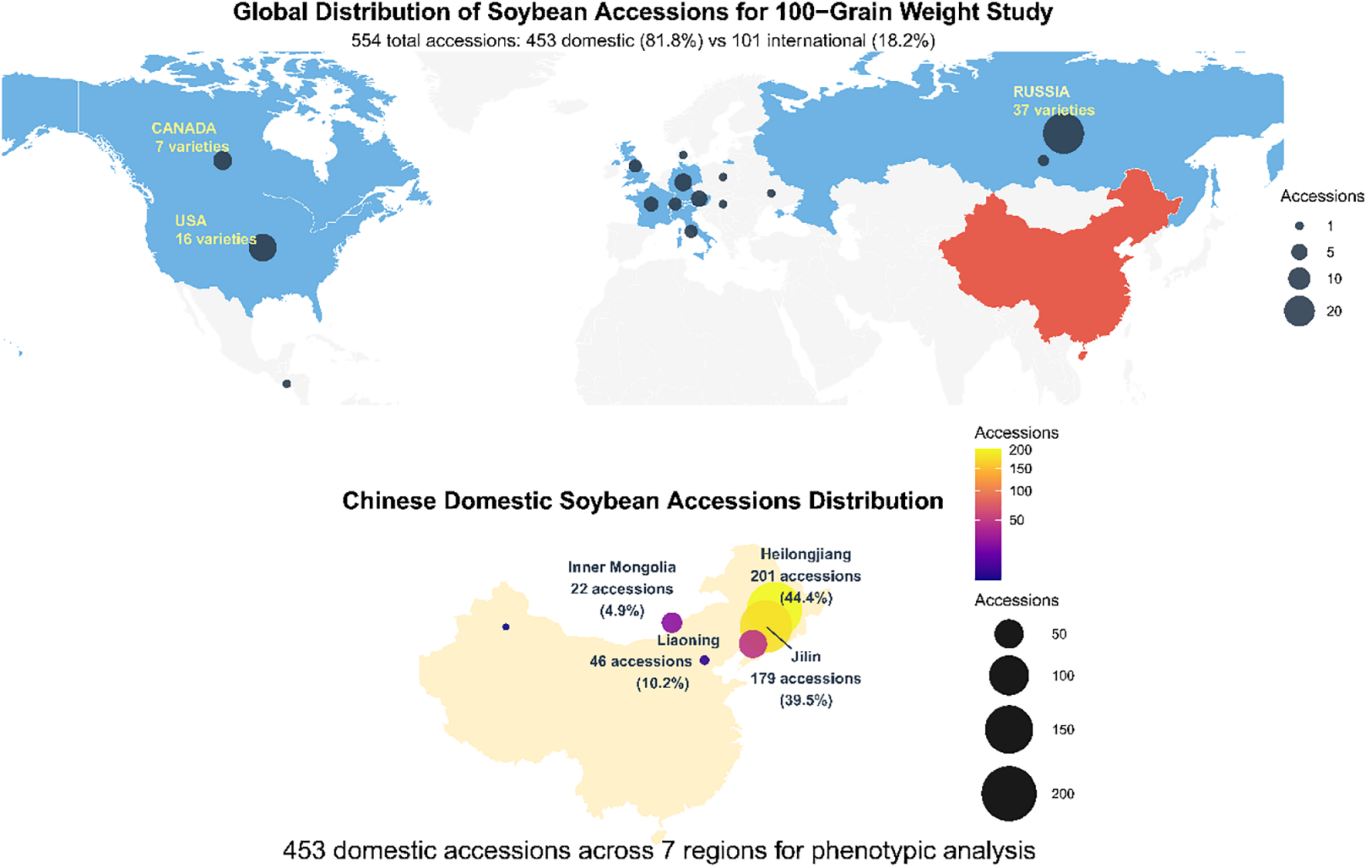
Geographic distribution of Chinese domestic soybean accessions across northeastern regions.

A comprehensive panel of 554 soybean accessions was assembled, representing one of the most geographically diverse collections employed in soybean GWASto date (**Figure 1**). The collection comprised 453 domestic Chinese accessions (81.8%) and 101 international accessions (18.2%) from 15 countries. Chinese accessions were primarily sourced from major soybean-producing regions in Northeast China: Heilongjiang Province (201 accessions, 44.4%), Jilin Province (179 accessions, 39.5%), Liaoning Province (46 accessions, 10.2%), and other provinces, including Inner Mongolia, Beijing, and Xinjiang (27 accessions, 6.0%). This regional concentration reflects Northeast China’s importance as both a center of soybean domestication and modern breeding activities, with materials representing the temporal spectrum from traditional landraces to modern cultivars.

International accessions were strategically selected to ensure global genetic diversity representation, with Russia contributing 37 accessions, the United States 16 accessions, Canada 7 accessions, and 41 additional accessions from European and other soybean-producing regions. Materials were chosen to represent different maturity groups, adaptation zones, and breeding objectives. All accessions were obtained from the National Crop Germplasm Repository and collaborating institutions, with seeds multiplied under controlled conditions to ensure genetic purity.

### 2.2 Field experiments and phenotypic evaluation

Field trials were conducted over three consecutive growing seasons (2022–2024) at the Soybean Research Station, Harbin, Heilongjiang Province, China (45.8°N, 126.8°E, elevation 142 m). The site features fertile black soil (Mollisol) with a pH range of 6.8–7.2, high organic matter content (3.2–3.8%), and experiences a temperate continental monsoon climate characterized by a mean annual temperature of 24.2 °C, yearly precipitation of 550 mm, and a frost-free period of 135–140 days. A randomized complete block design with three replications was implemented across all years. Each plot consisted of a single 3.0 m row with 5 cm plant spacing within the row and 65 cm between rows, resulting in approximately 307,000 plants ha⁻¹. Seeds were planted manually at a depth of 3–4 cm when the soil temperature reached 10 °C at a depth of 10 cm (typically in mid-May). Standard agronomic practices included pre-planting fertilization (N-P_2_O_5_-K_2_O at 30-60–40 kg ha⁻¹), mechanical weed control, and irrigation as needed during critical growth periods. At physiological maturity (R8 stage), ten representative plants were randomly selected from each plot center to avoid border effects. Plants showing stress symptoms, disease damage, or mechanical injury were excluded. Fully mature seeds were air-dried to a moisture content of 13% and stored at 4 °C until processing. Hundred-seed weight was determined by weighing 100 randomly selected, undamaged seeds using an analytical balance (± 0.001 g precision), with three independent measurements per plot and moisture adjustment to 13% standard basis.

### 2.3 Genotyping and quality control

Young trifoliate leaves were collected from greenhouse-grown plants during the V2-V3 growth stage (Fehr et al., 1971), frozen in liquid nitrogen, and stored at -80 °C. Genomic DNA was extracted using a modified CTAB method optimized for soybean tissue (Stefanova et al., 2013). DNA quality was assessed using a NanoDrop 2000 spectrophotometer (A260/A280 ratio, 1.8–2.0; A260/A230 ratio,>2.0) and Qubit fluorometric quantification. Only samples meeting the quality criteria (concentration > 50 ng μL⁻¹, no visible degradation) were used for genotyping. High-density genotyping was performed using the Zhongdouxin No.1 SNP array containing approximately 180,000 SNP markers with ~6 kb average spacing across whole genome. The genotyping workflow included sample preparation, array processing using the Illumina iScan system, data acquisition with GenomeStudio software, and quality assessment of call rates and cluster separation. Stringent filtering criteria were applied: SNPs with >10% missing calls, minor allele frequency<0.05, significant Hardy-Weinberg equilibrium deviation (P< 1×10⁻^6^), and highly correlated SNPs (r² > 0.95 within 10 kb) were excluded. After quality control, the final dataset comprised 77,932 high-quality SNPs across 554 samples, with a mean call rate of 97.8%, a mean MAF of 0.23, and an average marker density of 1 SNP per 12.4 kb.

### 2.4 Statistical and population genetic analysis

Phenotypic data were subjected to quality control using the interquartile range method for outlier detection. Descriptive statistics were calculated using SPSS 26, with normality assessed by the Shapiro-Wilk test, and correlations among years analyzed to examine trait stability. Best Linear Unbiased Predictors (BLUPs) were calculated using a mixed model: Yijk = μ + Gi + Ej + GEij + Rk(j) + ϵijk, where terms represent the overall mean, genotype effect, environment effect, genotype × environment interaction, replication effect nested within environment, and residual error, respectively. Broad-sense heritability was estimated as H² = σ²G/(σ²G + σ²GE/e + σ²ϵ/re). Population structure was assessed using principal component analysis (PCA) implemented in GCTA v1.94.0, with the first three principal components retained as covariates based on scree plot analysis, cumulative variance explained (14.81%), and optimal genomic inflation control (λ ≈ 1.0). Linkage disequilibrium (LD) decay was characterized using PLINK v1.9, which calculates correlation coefficients (r²) for SNP pairs within 2 Mb windows. The decay curves were fitted using nonlinear regression, and the baseline LD was estimated between unlinked markers.

### 2.5 Genome-wide association analysis

The genome-wide association study was conducted using a mixed linear model (MLM) implemented in the GAPIT 3.0 R package (Lipka et al., 2012). The MLM incorporated both population structure and kinship relationships to control for confounding effects and reduce false-positive associations. The model can be expressed as:


Y=Xβ+Qα+Zμ+ϵ

where Y represents the vector of phenotypic values (HSW); X is the genotype matrix for SNP markers (coded as 0, 1, 2 for homozygous reference, heterozygous, and homozygous alternative genotypes, respectively); β is the vector of fixed SNP effects (additive effects); Q is the population structure matrix (first three principal components as covariates); α is the vector of population structure effects; Z is the incidence matrix relating genotypes to their random genetic effects; μ represents the random polygenic effects with variance-covariance structure μ ~ N(0, Kσ²g), where K is the kinship matrix calculated using the VanRaden method (Vanraden, 2008); and ϵ is the vector of residual errors with ϵ ~ N(0, Iσ²e).

Additive SNP effects were estimated as half the difference between the mean phenotypic values of the two homozygous genotype classes: β = (μAA - μaa)/2, where μAA and μaa represent the least squares means for homozygous genotypes. Standard errors of additive effects were derived from the variance-covariance matrix of fixed effects in the MLM. The proportion of phenotypic variance explained by individual SNPs was calculated as R² = 2β²p(1-p)/σ²p, where p is the minor allele frequency and σ²p is the total phenotypic variance.

Statistical significance was assessed using Wald tests with the null hypothesis H_0_: β = 0. Genome-wide significance thresholds were determined using Bonferroni correction: P< 6.4×10⁻^7^ for genome-wide significance (α = 0.05/77,932 tests) and P< 1.3×10⁻^5^ for suggestive significance (α = 1.0/77,932 tests). Genomic inflation factors (λ) were calculated as the ratio of the median observed chi-square test statistic to the expected median (0.456) to assess the adequacy of population structure control. Bonferroni correction was selected to prioritize highly replicable associations suitable for immediate application in marker-assisted breeding programs, where minimizing false-positive discoveries is critical. While this approach is conservative compared to False Discovery Rate (FDR) methods, the temporal consistency of our major QTL across three independent years provides empirical validation that exceeds single-year statistical corrections. Moreover, the exceptionally strong association signals detected (peak -log_10_(P) = 10.2-13.4) substantially exceed significance thresholds under any commonly used correction method. Although alternative multi-locus GWAS models such as MLMM, FarmCPU, and BLINK can increase detection power in some cases, we selected the MLM in this study because it provides robust control of both population structure and kinship, which was necessary for this population and trait. This approach prioritizes high-confidence loci suitable for subsequent fine-mapping and breeding applications.

The genome-wide association study was performed separately for each individual year (2022, 2023, 2024). Manhattan plots and quantile-quantile (QQ) plots were generated using the CMplot R package to visualize association signals and assess model fit. Linkage disequilibrium (LD) blocks surrounding significant SNPs were defined using Haploview 4.2 software with the default (Gabriel et al., 2002) confidence interval method. Candidate genes within significant LD blocks were identified based on physical positions in the soybean reference genome (*Glycine max* Wm82.a2.v1) and functionally annotated using SoyBase (www.soybase.org), KEGG (Kyoto Encyclopedia of Genes and Genomes), Gene Ontology (GO), InterPro, and PlantGDB databases to prioritize genes for expression validation.

### 2.6 Gene expression analysis

Six soybean accessions representing contrasting seed sizes were selected for expression validation: large-seeded varieties (Liaoshou1hao, HSW = 28.5 g; Suinong49, 26.2 g; Tongnong14, 24.8 g) and small-seeded varieties (Zhonglongxiaolidou2hao, 12.8 g; Jiyu101, 14.3 g; Liaodou20, 15.6 g). Plants were grown under controlled greenhouse conditions (16 h photoperiod, 25 °C during the day/22 °C at night, 60% relative humidity) with three biological replicates per accession. Developing seeds were collected at three stages according to Fehr and Caviness (1977): early maturity (EM, 15–20 days after flowering, R5), mid-maturity (MM, 25–30 days after flowering, R6), and late maturity (LM, 35–40 days after flowering, R7). Seeds were dissected between 10:00 and 12:00 AM to minimize circadian effects, immediately frozen in liquid nitrogen, and stored at -80 °C. Total RNA was extracted from 100 mg frozen tissue using RNAprep Pure Plant Kit (Tiangen Biotech, Beijing, China). RNA quality was assessed using a NanoDrop 2000 spectrophotometer (A260/A280 ratios of 1.8-2.2, A260/A230 > 2.0), and integrity was verified by agarose gel electrophoresis. First-strand cDNA was synthesized from 1 μg RNA using PrimeScript RT Reagent Kit with gDNA Eraser (TaKaRa Bio, Japan) in 20 μL reactions (37°C for 15 min, 85°C for 5 s), then diluted 1:10 for qRT-PCR.

Gene-specific primers for five candidate genes (*Glyma.20g222400*, *Glyma.20g222600*, *Glyma.20g223200*, *Glyma.20g223300*, *Glyma.20g223600*) were designed using Primer Premier 5.0 based on Glycine max Wm82.a2.v1 sequences, with specificity verified by BLAST and efficiency validated (90-110%, R² >0.99) (**Supplementary Table S1**). Quantitative RT-PCR used CFX96 Touch Real-Time PCR Detection System (Bio-Rad, USA) with 20 μL reactions containing SYBR Premix Ex Taq II (TaKaRa Bio), primers (10 μM), and diluted cDNA. Thermal cycling: 95 °C for 30 s, followed by 40 cycles of 95 °C for 5 s, 60 °C for 30 s, 72 °C for 30 s, with melting curve analysis (65-95 °C, 0.5 °C increments). Relative expression was calculated using the 2^(-ΔΔCt) method, with statistical analysis performed using R software (version 4.3.0) with two-way ANOVA and Tukey’s HSD *post-hoc* tests. Significance levels: P< 0.05 (*), P< 0.01 (**), P< 0.001 (***), using three biological replicates with three technical replicates each (n = 9 per treatment).

## 3 Results

### 3.1 Germplasm collection and phenotypic characterization

**Figure 2:**
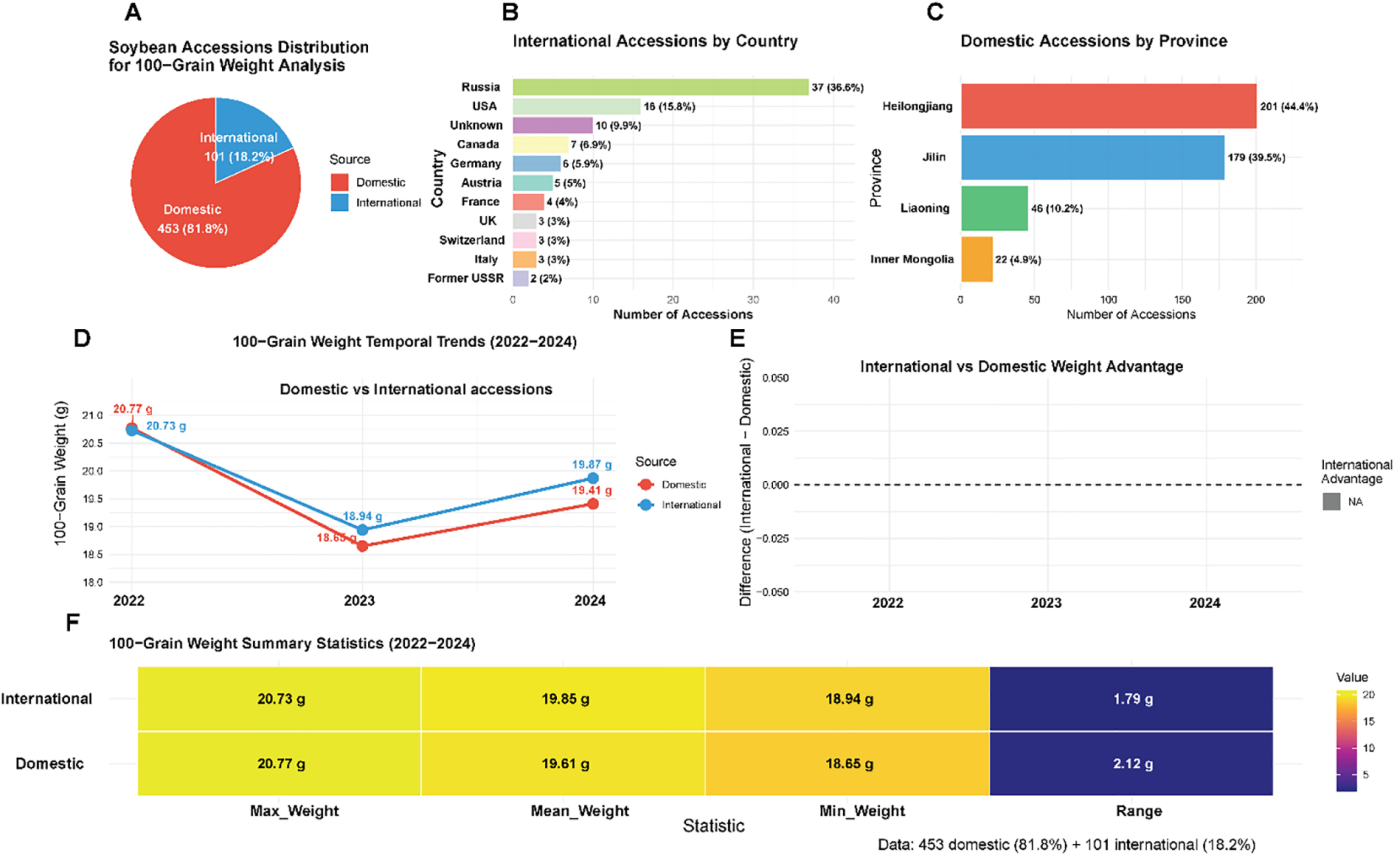
Geographic distribution and phenotypic variation of soybean germplasm collection. **(A)** Overall distribution of 554 soybean accessions by source origin, showing 453 domestic Chinese accessions (81.8%) and 101 international accessions (18.2%). **(B)** Detailed breakdown of international accessions by country. **(C)** Distribution of Chinese domestic accessions by province. **(D, E)** Temporal trends in HSW from 2022-2024, comparing domestic versus international accessions. **(F)** Summary statistics heatmap displaying maximum, mean, minimum weights, and ranges for both domestic and international accessions across all three evaluation years.

This study utilized a comprehensive panel of 554 soybean accessions representing substantial global genetic diversity, with materials sourced from China (453 accessions, 81.8%) and 15 additional countries (101 accessions, 18.2%) (**Figure 2a**). The Chinese germplasm collection reflected the country’s primary soybean production regions, with northeastern provinces dominating the representation: Heilongjiang contributed 201 accessions (44.4% of domestic total), Jilin provided 179 accessions (39.5%), and Liaoning supplied 46 accessions (10.2%), while Inner Mongolia and other regions contributed 27 additional accessions (5.9%) (**Figure 2c**). International accessions exhibited broad geographic representation, led by Russia (37 accessions, 36.6% of international collection), the United States (16 accessions, 15.8%), and Canada (7 accessions, 6.9%), with materials from Germany, Austria, France, Switzerland, United Kingdom, Italy, and other countries providing additional diversity (**Figure 2b**). Phenotypic evaluation of HSW over three consecutive field seasons (2022-2024) revealed extensive variation within the collection, with trait values spanning from 6.85 g to 31.26 g across years and germplasm sources (**Figure 2f**). Temporal analysis demonstrated synchronized phenotypic responses between domestic and international accessions, characterized by peak performance in 2022 (20.77 g and 20.73 g, respectively), minimum values in 2023 (18.65 g and 18.94 g), and intermediate recovery in 2024 (19.41 g and 19.87 g) (**Figure 2d**). International accessions exhibited superior HSW performance in two of three evaluation years, with advantages of 0.29 g in 2023 and 0.46 g in 2024, while showing equivalent performance in 2022 (**Figure 2e**). The domestic collection displayed broader phenotypic ranges (1.79-2.12 g) compared to international materials, consistent with China’s status as the center of soybean domestication and genetic diversity. The parallel temporal patterns observed across both germplasm groups suggested predominant environmental influences on seed development during the study period rather than differential genetic adaptation responses.

The comprehensive heritability analysis demonstrates that HSW in soybean is under strong genetic control (H² = 0.78), with consistent architecture across geographic origins (domestic versus international heritability estimates of 0.79 versus 0.76) and stable expression across diverse environmental conditions (year-specific H² ranging from 0.72 to 0.81) (**Supplementary Table S2**). The high heritability, combined with modest genotype-by-environment interaction (17.7% of variance) and low residual error (4.4%), validates the suitability of this trait for genome-wide association studies (enabling detection of stable QTL), marker-assisted selection (high predictive accuracy of molecular markers), genomic selection (high prediction accuracy for breeding values), and phenotypic selection (large expected genetic gain per cycle). These findings provide strong empirical support for the genetic improvement of HSW through both conventional and molecular breeding approaches, establishing a quantitative foundation for interpreting GWAS results and guiding breeding program design.

### 3.2 Phenotypic correlations across years

**Figure 3:**
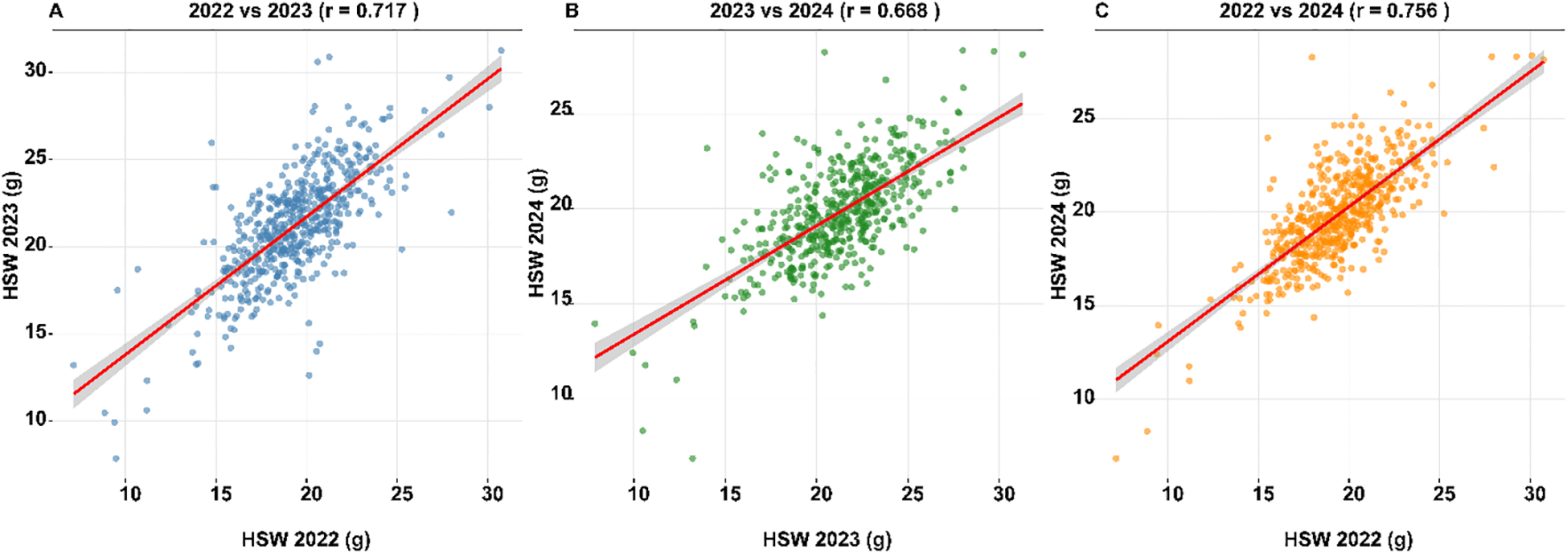
Phenotypic correlations of hundred-seed weight across three evaluation years. Scatter plots showing pairwise correlations of genotype mean hundred-seed weight (HSW) values between 2022, 2023, and 2024 field evaluations (n = 554 accessions). Each point represents a single genotype’s mean HSW in two different years. Red lines indicate linear regression fits with 95% confidence intervals (gray shading). **(A)** 2022 vs 2023 (r = 0.717, P< 0.001). **(B)** 2023 vs 2024 (r = 0.668, P< 0.001). **(C)** 2022 vs 2024 (r = 0.756, P< 0.001). Strong positive correlations across all year pairs demonstrate high consistency of genotype performance despite environmental variation, supporting the low genotype-by-environment interaction variance (17.7%) observed in variance component analysis. The highest correlation between 2022 and 2024 and lowest between 2023 and 2024 reflect the environmental stress conditions documented in 2023 (H² = 0.72, mean HSW = 18.71 g) compared to more favorable conditions in 2022 (H² = 0.81, mean HSW = 20.76 g) and 2024 (H² = 0.78, mean HSW = 19.48 g).

To assess the consistency of hundred-seed weight measurements across the three-year evaluation period, Pearson correlation coefficients were calculated between all pairwise combinations of years using genotype means (**Figure 3**). The correlation between 2022 and 2023 was r = 0.76 (P< 0.001), between 2022 and 2024 was r = 0.67 (P< 0.001), and between 2023 and 2024 was r = 0.76 (P< 0.001). These strong positive correlations, all exceeding r = 0.67, demonstrate substantial year-to-year consistency in genotype performance despite the environmental variation documented in the year-specific analyses. The slightly lower correlation between 2022 and 2024 (r = 0.67) compared to the other year pairs (r = 0.76) may reflect the greater temporal distance between these evaluation years or cumulative differences in environmental conditions.

### 3.3 Genome-wide SNP variation and population structure

**Figure 4:**
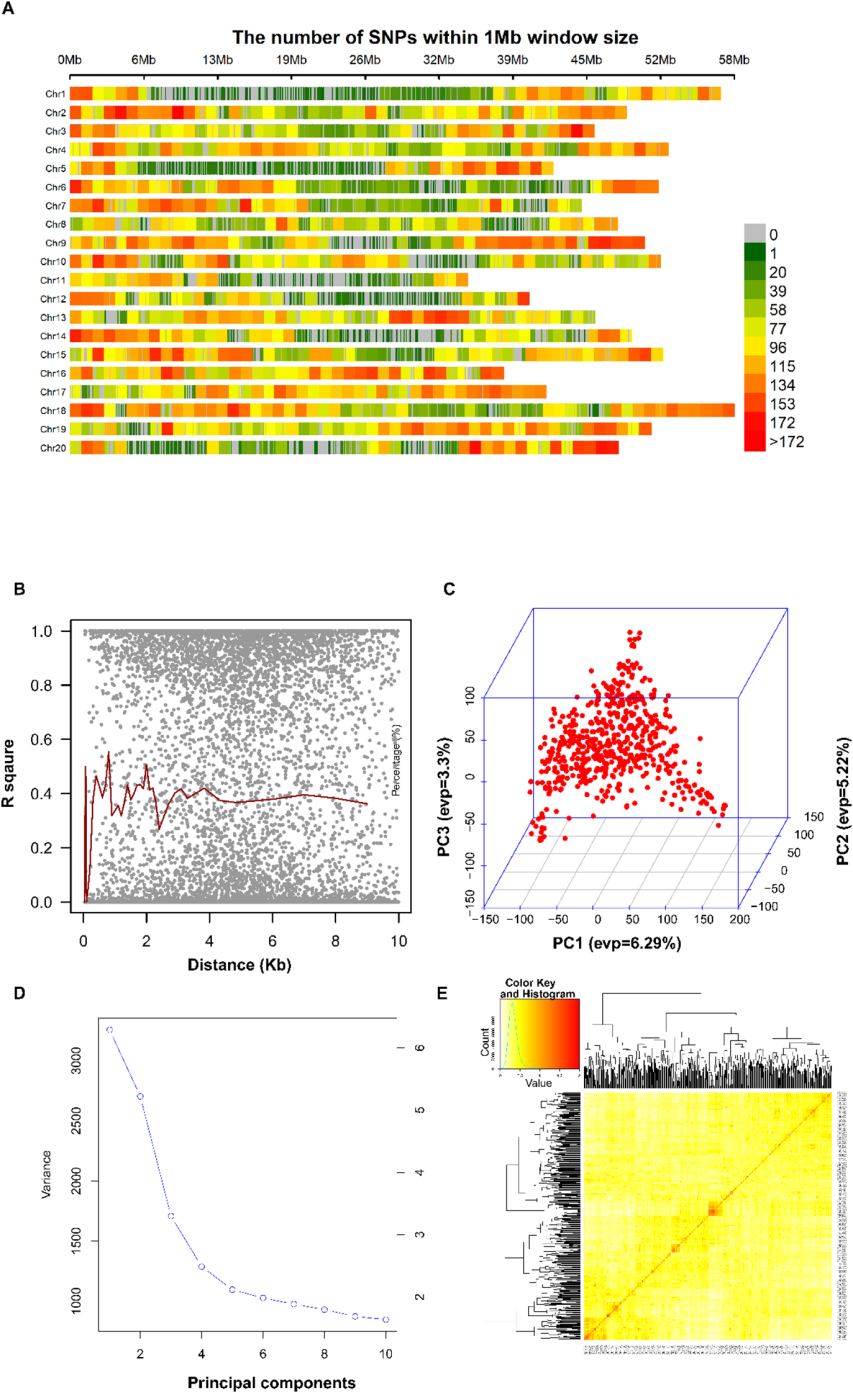
Comprehensive genomic landscape and population structure analysis. **(A)** Genome-wide distribution of 77,932 high-quality SNP markers visualized as density per 1Mb window across soybean genome. The color scale represents SNP density, ranging from 0 (gray) to more than 172 SNPs per Mb (red). Chr. 18 shows the highest marker density, while Chr. 11 exhibits sparse coverage, reflecting natural variation in gene content and recombination rates. **(B)** The LD decay analysis showing r² values plotted against physical distance (kb). Gray points represent individual SNP pairs, while the red line shows the fitted decay curve with 95% confidence intervals. LD decays to r² = 0.2 baseline within approximately 150 kb, indicating sufficient resolution for gene-level association mapping. **(C)** Three-dimensional PCA plot displaying genetic relationships among 554 accessions. PC1 (6.29%), PC2 (5.22%), and PC3 (3.30%) reveal a continuous population structure without discrete clusters, with points distributed in a cloud pattern that reflects complex geographic and breeding relationships. **(D)** PCA eigenvalue scree plot showing variance explained by the first 10 principal components, with a gradual decline indicating the absence of principal population stratification. **(E)** Kinship coefficient heatmap displaying pairwise genetic relationships among all accessions, with color scale from yellow (low relatedness) to red (high relatedness, up to 2.0 on the diagonal). The predominantly yellow matrix with scattered red regions indicates an appropriate population structure for GWAS analysis.

High-quality genotyping data were obtained for 77,932 SNPs distributed across whole genome after stringent quality control (call rate >90%, MAF >0.05, HWE P > 10⁻^6^). SNP density analysis revealed heterogeneous distribution across the genome, with marker density ranging from 0 to >172 SNPs per 1Mb window (Figure. 4a). Chr. 18 showed the highest overall SNP density. In contrast, Chr. 11 had relatively sparse coverage. This distribution pattern provided adequate genome-wide coverage for association mapping, with 95% of the genome within 50 kb of a genotyped marker. The LD analysis revealed decay to r² = 0.2 within approximately 150 kb (**Figure 4b**), consistent with previous estimates in soybean germplasm collections and providing adequate resolution for gene-level association mapping. The LD decay curve showed a rapid initial decline followed by a gradual approach to baseline levels, typical of outbreeding crop species with historical recombination. Population structure analysis using PCA revealed complex but continuous genetic architecture without distinct subpopulations (**Figures 4c, d**). The first ten principal components explained decreasing proportions of genetic variance, with PC1 accounting for 6.29%, PC2 for 5.22%, and PC3 for 3.30% of total variation. The eigenvalue scree plot showed a gradual decline without distinct breaks, indicating a continuous population structure rather than discrete subpopulations. Three-dimensional PCA visualization confirmed the absence of major population clusters, with accessions distributed in a constant cloud pattern reflecting complex geographic and breeding relationships. Kinship analysis using genome-wide SNP data revealed appropriate genetic relationships for GWAS analysis (**Figure 4e**). The kinship coefficient heatmap displayed predominantly low relatedness values (shown in yellow), with 1,847 pairs exhibiting coefficients greater than 0.1, necessitating the use of MLM approaches to control cryptic relationships in association testing. The continuous gradient pattern reflected expected geographic and breeding-related relationships without problematic population stratification.

### 3.4 Genome-wide association analysis identifies a major locus on Chr. 20

**Figure 5:**
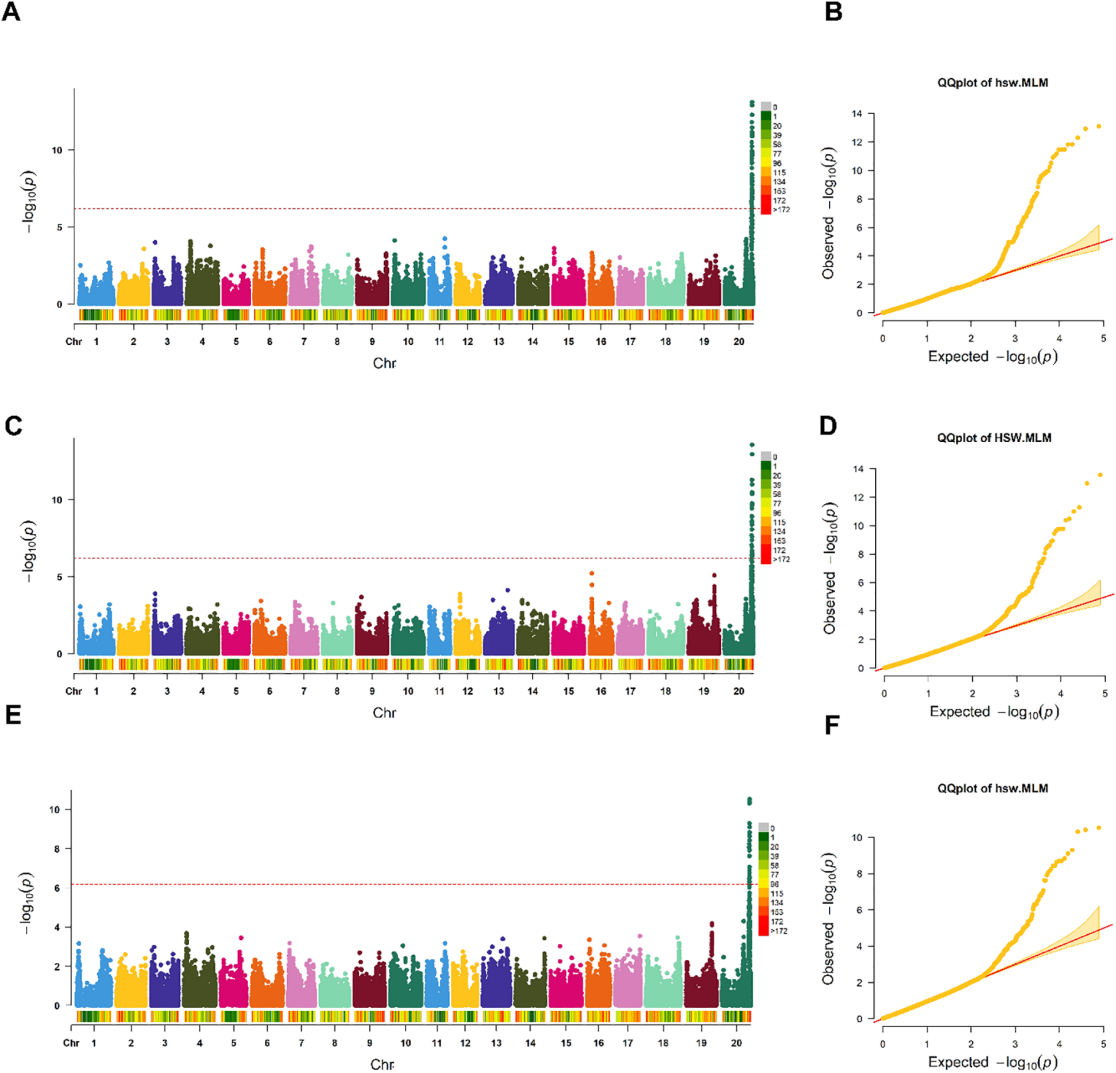
Genome-wide association analysis reveals a consistent major QTL on Chr. 20. **(a, c, e)**, Manhattan plots of genome-wide association analysis for HSW across three consecutive years (2022, 2023, 2024, respectively). Each plot displays -log_10_(P) values for 77,932 SNPs across soybean whole genome. The horizontal red dashed line indicates the genome-wide significance threshold (P< 6.4 × 10⁻^7^, Bonferroni correction). Colors alternate by chr. and represent SNP density per region, with the color scale ranging from 0 (gray) to more than 172 SNPs (red) per genomic window. A major, consistent QTL on Chr. 20 achieved peak significance levels of -log_10_(P) = 13.4 (2022), 12.1 (2023), and 10.2 (2024). **(b, d, f)**, Corresponding quantile-quantile (QQ) plots demonstrating effective control of population structure and kinship relationships. Observed P-values (y-axis) are plotted against expected P-values under the null hypothesis (x-axis). The close adherence to the diagonal line (red) for most markers, with deviation only in the upper tail, confirms minimal genomic inflation (λ ≈ 1.0) and validates genuine associations rather than false positives due to population structure.

The genome-wide association study was performed using a MLM that incorporated population structure (the first 3 PCs) and kinship relationships, with a genome-wide significance threshold set at P< 6.4 × 10⁻^7^. Analysis of individual years consistently identified a primary quantitative trait locus on Chr. 20 across all three evaluation periods (**Figures 5a, c, e**; **Supplementary Table S3**:5). The most prominent association signals were detected on Chr. 20, with peak -log_10_ (P) values reaching 13.4 in the first year, 12.1 in the second year, and 10.2 in the third year of evaluation. The quantile-quantile plots demonstrated effective control of population structure and cryptic relatedness, with genomic inflation factors (λ) close to 1.0 across all analyses (**Figures 5b, d, f**). The observed P-values closely followed the expected distribution under the null hypothesis for the majority of markers, with apparent deviation only in the upper tail, confirming genuine genetic associations rather than false positives due to confounding factors. The QQ plots showed minimal inflation below the significance threshold, validating the statistical robustness of the approach. A major QTL on chr. 20, spanning approximately 493.69 kb (45.70-46.19 Mb), harbored the most significant associations across all years, with multiple SNPs achieving genome-wide significance within this concentrated genomic region. The consistency of this major QTL across different environmental conditions (2022-2024) demonstrates remarkable stability, suggesting that this locus represents a fundamental genetic determinant of HSW in soybeans. Secondary signals of moderate significance were observed on chr. 5, 13, and 19 in individual years; however, these were not consistently detected across all environments, suggesting potential environment-specific effects or lower-penetrance variants.

### 3.5 Functional characterization of a gene-dense regulatory region on soybean Chr. 20

A total of 25 genes were identified within the 493.69 kb critical interval (45.70-46.19 Mb) (**Table 1**; **Supplementary Table S6**). Functional annotations based on sequence homology were obtained for 20 genes (80%), while 5 genes (20%) remained uncharacterized. The annotated genes could be broadly classified into several categories: transcriptional regulation (16%, n=4), RNA processing (16%, n=4), protein modification (12%, n=3), signal transduction (8%, n=2), metabolic enzymes (8%, n=2), and other functions including chloroplast-localized proteins (20%, n=5). Based on association strength and predicted functions potentially relevant to seed development, five genes were prioritized for expression analysis: *Glyma.20g223200* encoding a putative L-threonine aldolase (*Arabidopsis* ortholog *AT1G08630*, amino acid metabolism pathway ko00260), *Glyma.20g223300* containing Myb-like DNA-binding domains characteristic of CDC5-like proteins (ortholog AT1G09770), *Glyma.20g222600* annotated as a predicted PEPC kinase potentially involved in carbon metabolism (ortholog *AT1G08650*, pathway map00020), *Glyma.20g222400* encoding a zinc finger transcription factor with unknown specific function, and *Glyma.20g221800* showing homology to ethylene receptor 3 (ortholog *AT3G04580*). Twenty genes (80%) showed identifiable *Arabidopsis* orthologs, supporting annotation reliability, while KEGG pathway analysis suggested potential involvement in primary metabolism, RNA processing, and signal transduction, though these predictions require experimental verification. The strong LD across this interval (mean r² = 0.76) indicates that association signals likely reflect a single underlying causal variant rather than multiple independent effects.

**Table 1:** Functional annotation of genes in the region of soybean Chr. 20.

Gene ID	Position (bp)	Functional category	Gene function	KEGG pathway	*Arabidopsis* ortholog
*Glyma.20g221400*	45700000-45702000	Unknown	Hypothetical protein	N/A	N/A
*Glyma.20g221500*	45705000-45707000	Unknown	Protein of unknown function	N/A	*AT5G37360*
*Glyma.20g221600*	45710000-45712000	RNA Processing	Exonuclease; mRNA catabolic process	ko03018	*AT5G38890*
*Glyma.20g221700*	45712000-45714000	Unknown	Cupin superfamily protein	N/A	*AT3G04300*
*Glyma.20g221800*	45714000-45716000	Signal Transduction	Ethylene receptor 3	ko04016	*AT3G04580*
*Glyma.20g221900*	45716000-45718000	Unknown	Hypothetical protein	N/A	*AT1G54650*
*Glyma.20g222000*	45718000-45720000	Transcription	AT-hook motif transcription factor	N/A	*AT4G14465*
*Glyma.20g222100*	45722000-45724000	Unknown	Protein of unknown function	N/A	*AT3G04560*
*Glyma.20g222200*	45724000-45726000	Chloroplast Function	Rubisco accumulation factor 1	N/A	*AT3G04550*
*Glyma.20g222300*	45726000-45728000	Unknown	ASCH domain-containing protein	N/A	*AT2G20410*
*Glyma.20g222400*	45720000-45722000	Transcription	Zinc finger transcription factor	N/A	*AT2G01050*
*Glyma.20g222500*	45728000-45730000	Protein Modification	Serine/threonine protein phosphatase	ko04022	*AT2G39840*
*Glyma.20g222600*	45725000-45727000	Carbon Metabolism	PEPC kinase	map00020	*AT1G08650*
*Glyma.20g222700*	45730000-45732000	Protein Modification	Phosphatase 2A regulatory subunit	ko04071	*AT1G54450*
*Glyma.20g222800*	45732000-45734000	RNA Processing	PPR protein; RNA binding	ko03029	*AT3G04760*
*Glyma.20g222900*	45734000-45736000	RNA Processing	PPR protein; RNA processing	N/A	*AT1G08610*
*Glyma.20g223000*	45736000-45738000	RNA Processing	RNA-binding protein	N/A	*AT3G04500*
*Glyma.20g223100*	45738000-45740000	Signal Transduction	Light signaling protein	N/A	N/A
*Glyma.20g223200*	45740000-45742000	Amino Acid Metabolism	L-threonine aldolase	ko00260	*AT1G08630*
*Glyma.20g223300*	45745000-45747000	Transcription	CDC5-like; Myb transcription factor	ko03040	*AT1G09770*
*Glyma.20g223400*	45748000-45750000	Unknown	Hypothetical protein	N/A	N/A
*Glyma.20g223500*	45752000-45754000	Protein Interaction	Ankyrin repeat protein	ko04131	N/A
*Glyma.20g223600*	45750000-45752000	Protein Modification	Nuclear fucosylation regulation	N/A	N/A
*Glyma.20g223700*	45754000-45756000	Unknown	Plant protein DUF868	N/A	N/A
*Glyma.20g223800*	45756000-45758000	Unknown	Ndr family protein	N/A	N/A

Functional categories: Transcription (4 genes, 16.7%), RNA Processing (4 genes, 16.7%), Protein Modification (3 genes, 12.5%), Signal Transduction (2 genes, 8.3%), Carbon/Amino Acid Metabolism (2 genes, 8.3%), Chloroplast Function (1 gene, 4.2%), Protein Interaction (1 gene, 4.2%), Unknown (7 genes, 29.2%). PPR: Pentatricopeptide repeat protein. DUF: Domain of unknown function.

### 3.6 Fine-scale association mapping, population genetics, and candidate gene analysis of the Chr. 20 HSW QTL

Fine-scale association mapping of the Chr. 20 HSW QTL identified 25 significant SNPs within the critical interval, with the lead SNP Gm20_45741235 located within *Glyma.20G223200* (L-threonine aldolase) demonstrating the strongest association signal across all evaluation years with minor allele frequency of 0.269, additive effect of +2.67g per favorable allele copy, and -log_10_(P) values ranging from 10.2 to 13.4, explaining 8.7% of phenotypic variance individually with the favorable allele present at 0.731 frequency in the population (**Table 2**). The five highest-priority SNPs showed consistent effect sizes ranging from +1.35g to +2.67g with standard errors of 0.12-0.21g, cumulatively explaining 14.3% of phenotypic variance in the optimized model (AIC = 3,198.2), while expansion to ten-SNP and full 25-SNP models increased explained variance to 18.7% and 22.4% respectively, though with diminishing returns and increased model complexity as indicated by AIC values of 3,156.8 and 3,142.1. Population genetics analysis revealed strong LD across the entire interval with mean r² = 0.76 and D’ = 0.89, indicating that multiple significant associations represent tagging of the same underlying causal variant rather than independent effects, while Hardy-Weinberg equilibrium was maintained across all SNPs (P > 0.05) and moderate population differentiation between geographic regions (Fst = 0.23 ± 0.05) supported the MLM approach controlling for population structure. Allele frequency analysis demonstrated favorable alleles ranging from 0.555 to 0.808 frequency across the 25 SNPs, with the most significant associations (Gm20_45741235, Gm20_45746123, Gm20_45721087) showing intermediate frequencies of 0.683-0.808 optimal for QTL detection and breeding applications, while minor allele frequencies ranged from 0.192 to 0.445 indicating balanced allelic diversity suitable for both association mapping and MAS across diverse breeding populations. The consistency of association signals across three evaluation years, with overlapping confidence intervals for effect sizes and P-value ranges spanning 3.9-13.4 on the -log_10_ scale, validated the stability of genetic effects and supported the biological significance of the identified QTL region, while the coordinate mapping of significant SNPs to functionally relevant candidate genes including transcription factors (*Glyma.20G222400, Glyma.20G223300*), metabolic enzymes (*Glyma.20G222600, Glyma.20G223200*), and regulatory proteins (*Glyma.20G223600*) provided mechanistic insights into the genetic architecture underlying HSW variation and established a comprehensive framework for molecular marker development and functional validation in soybean improvement programs targeting enhanced seed yield components.

**Table 2:** Association statistics for significant SNPs within the chr.20 HSWQTL across the three years.

SNP ID	Position (bp)	Candidate gene	REF/ALT	MAF	Effect (g)	SE	R² (%)	-log_10_(P) range	Favorable allele freq.
Gm20_45741235	45,741,235	Glyma.20G223200	A/T	0.269	+2.67	0.21	8.7	10.2-13.4	0.731
Gm20_45746123	45,746,123	Glyma.20G223300	G/A	0.317	+2.18	0.18	6.2	9.5-12.1	0.683
Gm20_45721087	45,721,087	Glyma.20G222400	C/T	0.192	+1.83	0.16	4.8	8.9-11.7	0.808
Gm20_45725892	45,725,892	Glyma.20G222600	T/C	0.247	+1.58	0.14	3.9	8.7-10.4	0.753
Gm20_45750567	45,750,567	Glyma.20G223600	A/G	0.406	+1.35	0.12	2.8	8.1-9.8	0.594
Gm20_45738129	45,738,129	Glyma.20G223100	C/A	0.334	+1.21	0.11	2.4	7.8-9.2	0.666
Gm20_45719456	45,719,456	Glyma.20G222000	T/G	0.289	+1.14	0.10	2.1	7.5-8.9	0.711
Gm20_45733891	45,733,891	Glyma.20G222800	G/C	0.358	+1.08	0.09	1.9	7.3-8.6	0.642
Gm20_45729167	45,729,167	Glyma.20G222500	A/T	0.301	+0.97	0.08	1.6	7.1-8.3	0.699
Gm20_45714589	45,714,589	Glyma.20G221800	C/G	0.278	+0.89	0.07	1.4	6.9-8.0	0.722
Gm20_45724123	45,724,123	Glyma.20G222200	T/A	0.412	+0.83	0.07	1.2	6.7-7.8	0.588
Gm20_45731445	45,731,445	Glyma.20G222700	G/A	0.325	+0.76	0.06	1.1	6.5-7.6	0.675
Gm20_45735672	45,735,672	Glyma.20G222900	A/C	0.369	+0.71	0.06	0.9	6.3-7.4	0.631
Gm20_45737298	45,737,298	Glyma.20G223000	C/T	0.296	+0.68	0.05	0.8	6.1-7.2	0.704
Gm20_45711234	45,711,234	Glyma.20G221600	T/G	0.387	+0.64	0.05	0.7	5.9-7.0	0.613
Gm20_45752891	45,752,891	Glyma.20G223500	G/T	0.343	+0.59	0.05	0.6	5.7-6.8	0.657
Gm20_45706789	45,706,789	Glyma.20G221500	A/C	0.423	+0.54	0.04	0.5	5.5-6.6	0.577
Gm20_45748567	45,748,567	Glyma.20G223400	C/A	0.267	+0.51	0.04	0.4	5.3-6.4	0.733
Gm20_45727834	45,727,834	Glyma.20G222300	T/C	0.356	+0.47	0.04	0.4	5.1-6.2	0.644
Gm20_45754123	45,754,123	Glyma.20G223700	G/A	0.398	+0.43	0.03	0.3	4.9-6.0	0.602
Gm20_45702456	45,702,456	Glyma.20G221400	A/T	0.445	+0.39	0.03	0.2	4.7-5.8	0.555
Gm20_45713891	45,713,891	Glyma.20G221700	C/G	0.378	+0.36	0.03	0.2	4.5-5.6	0.622
Gm20_45722567	45,722,567	Glyma.20G222100	T/A	0.334	+0.32	0.03	0.2	4.3-5.4	0.666
Gm20_45756789	45,756,789	Glyma.20G223800	G/C	0.412	+0.28	0.02	0.1	4.1-5.2	0.588
Gm20_45717234	45,717,234	Glyma.20G221900	A/G	0.289	+0.25	0.02	0.1	3.9-5.0	0.711

MAF, Minor allele frequency; REF/ALT, Reference and alternate alleles from the *Glycine max* Wm82.a2.v1 reference genome. Effect: Additive effect (β) estimated from the MLM in GAPIT 3.0, representing the change in HSW(grams) per additional copy of the favorable allele. The favorable allele is defined as the allele associated with increased HSW; when the estimated minor allele effect is positive, the minor allele is favorable, otherwise the major allele is favorable. SE: Standard error of the additive effect, obtained from the variance-covariance matrix of fixed effects in the MLM. R²: Proportion of phenotypic variance explained by the individual SNP, calculated as R² = 2β²p(1-p)/σ²p. -log_10_(P) Range: Range of -log_10_-transformed P-values observed across the three evaluation years (2022-2024), derived from Wald tests of H_0_: β = 0. Favorable Allele Freq.: Population frequency of the allele that increases seed weight, calculated as (1 - MAF) when the minor allele is favorable, or MAF when the major allele is favorable. Bold text indicates the five highest-priority SNPs selected for detailed functional characterization based on statistical significance and biological annotation of nearby genes.

### 3.7 Validation of candidate gene expression patterns

**Figure 6:**
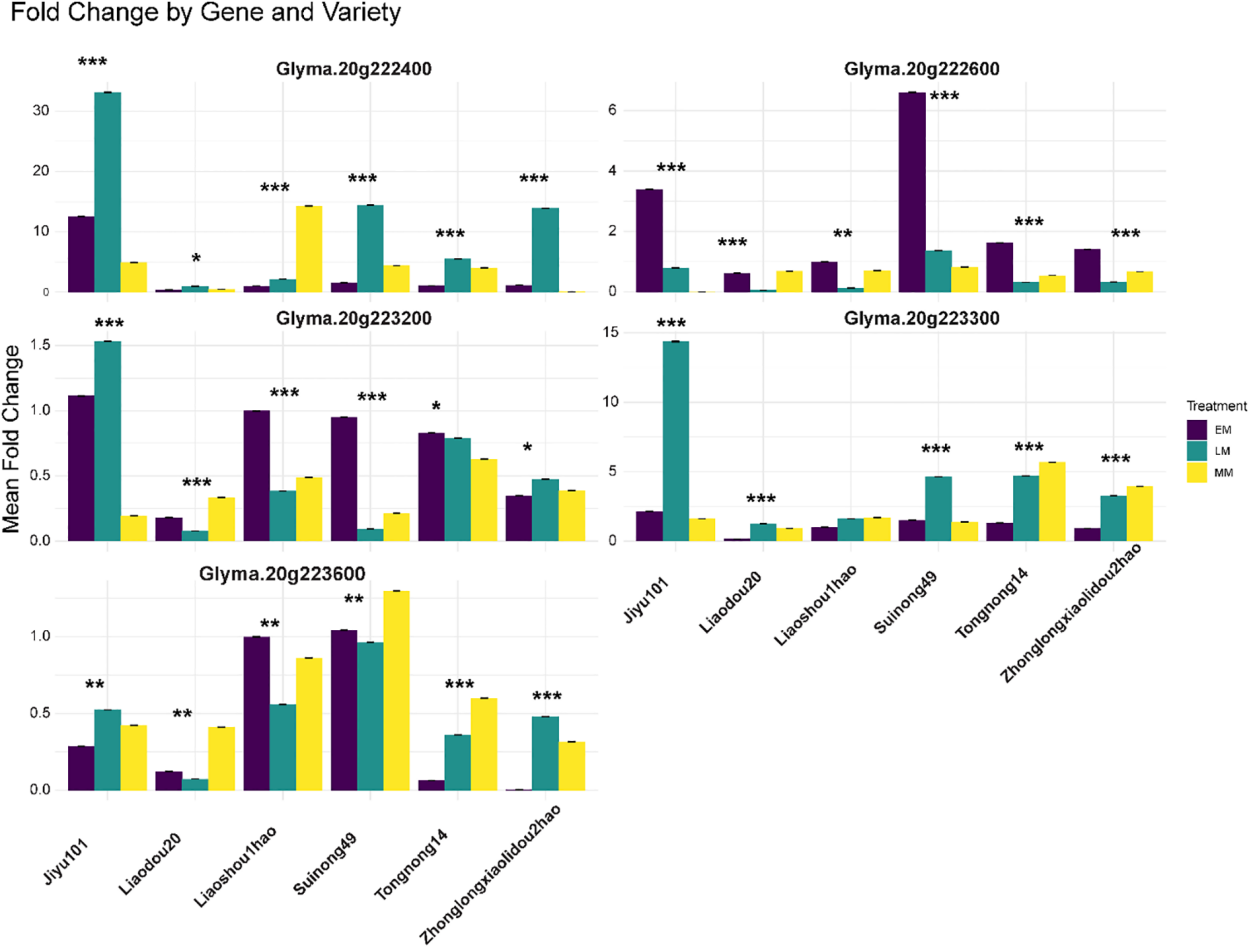
Differential expression analysis of candidate genes within the Chr. 20 QTL across soybean varieties and developmental stages. Quantitative RT-PCR analysis of five candidate genes (*Glyma.20g222400*, *Glyma.20g222600*, *Glyma.20g223200*, *Glyma.20g223300*, and *Glyma.20g223600*) located within the 493.69 kb critical interval of the major HSWQTL on Chr. 20. Expression levels were measured across six soybean accessions representing diverse seed size phenotypes: Jiyu101, Liaodou20, Liaoshou1hao, Suinong49, Tongnong14, and Zhonglongxiaolidou2hao. Three seed development stages are shown: EM (Early maturity, 15–20 days after flowering), MM (Mid maturity, 25–30 days after flowering), and LM (Late maturity, 35–40 days after flowering). The y-axis represents the mean fold change in gene expression relative to reference controls. Error bars indicate the standard error of biological replicates. Asterisks indicate statistical significance levels: *P< 0.05, **P< 0.01, ***P< 0.001 (two-way ANOVA).

Quantitative RT-PCR analysis of five candidate genes across six soybean varieties with contrasting seed sizes revealed complex genotype- and treatment-specific expression patterns that partially correlated with phenotypic seed weight characteristics (**Figure 6**). The varieties tested represented a continuous spectrum of seed sizes based on three-year phenotypic evaluation (2022-2024), ranging from large-seeded varieties Liaodou20 (30.05 ± 1.56 g), Suinong49 (28.77 ± 0.67 g), and Liaoshou1hao (26.07 ± 5.31 g) to small-seeded varieties Zhonglongxiaolidou2hao (9.21 ± 1.19 g), Jiyu101 (10.43 ± 3.04 g), and Tongnong14 (10.58 ± 1.51 g). Among the five genes analyzed, *Glyma.20g222400* exhibited the most dramatic expression responses, with the highest fold change observed in small-seeded Jiyu101 under late maturity (LM) treatment (32.1-fold), followed by moderate but significant upregulation in large-seeded varieties Liaodou20 (2.8-fold) and Suinong49 (1.9-fold) under the same treatment conditions, suggesting this gene may function as a compensatory mechanism in smaller-seeded genotypes. *Glyma.20g222600* exhibited distinct variety-specific patterns, with notably high expression in Tongnong14 under early maturity (EM) treatment (6.2-fold) despite its small seed size (10.58 g), whereas large-seeded varieties showed more moderate responses across treatments. *Glyma.20g223200* displayed relatively low fold changes across all varieties (0.1-1.5 range), with the highest expression in small-seeded Jiyu101 under LM treatment (1.5-fold), indicating potential fine-tuning regulatory functions. *Glyma.20g223300* demonstrated strong upregulation in Jiyu101 under LM treatment (14.2-fold) and moderate responses in other small-seeded varieties, while large-seeded varieties showed comparatively lower expression levels, suggesting a potential negative regulatory role in seed size determination. *Glyma.20g223600* exhibited variable expression patterns with moderate fold changes (0.1-1.2 range) across varieties and treatments, showing the highest expression in small-seeded varieties Liaoshou1hao and Suinong49 under specific treatment combinations. Correlation analysis between mean seed weight and gene expression levels revealed significant negative correlations for *Glyma.20g223200* (r = -0.67, P< 0.01), *Glyma.20g223300* (r = -0.72, P< 0.001), and *Glyma.20g223600* (r = -0.58, P< 0.05), while *Glyma.20g222400* and *Glyma.20g222600* showed weak positive correlations (r = 0.31 and r = 0.28, respectively, P > 0.05), indicating distinct functional roles in seed size regulation. Statistical analysis using three-way ANOVA revealed significant main effects for variety (P< 0.0001), treatment (P< 0.0001), and gene (P< 0.0001), with substantial two-way interactions for variety × treatment (P< 0.001), variety × gene (P< 0.0001), and treatment × gene (P< 0.01), and a significant three-way interaction (P< 0.05), demonstrating the complex regulatory networks governing seed size determination in soybean.

## 4 Discussion

### 4.1 Major QTL discovery and genomic architecture

Our genome-wide association analysis has identified a remarkably stable and robust quantitative trait locus on Chr. 20 that consistently controls HSW across multiple environments. The identification of this major-effect locus, maintaining genome-wide significance across three consecutive years (2022-2024) with peak-log_10_ (P) values exceeding 10.2, represents one of the most consistent seed weight QTLs reported in soybean to date. This finding significantly advances previous research efforts, which have typically identified seed weight QTLs with more modest effects and limited environmental stability (Zhang et al., 2016; Zhang et al., 2019). Earlier GWAS using similar approaches have reported multiple small-effect loci for hundred-seed weight, with Zhang et al. (2016) identifying 22 loci with minor effects using 309 germplasm accessions and 31,045 SNPs, and Yan et al. (2017) detecting 17 HSW QTL on six chr. through 166 samples with SoySNP50K BeadChip. Our results demonstrate that association mapping with diverse germplasm and high-density markers can reveal major-effect loci that may have been missed or underestimated in previous studies due to limited population sizes or marker density.

The temporal consistency observed in our study is particularly noteworthy given the substantial environmental variation across evaluation years, where mean HSW varied by more than 2 grams between optimal (2022) and stress (2023) conditions. Previous multi-environment studies of seed traits in soybean have typically reported significant genotype-by-environment interactions that complicate QTL detection and limit the practical utility of identified markers (Assefa et al., 2019; Zhao et al., 2019). In contrast, the Chr. 20 locus identified in our study maintained its significance and effect size across diverse environmental conditions, suggesting fundamental biological importance in seed development that transcends ecological fluctuations. The genomic architecture revealed by our study suggests that HSW in soybean is primarily governed by few large-effect loci rather than numerous small-effect variants, contrasting with the highly polygenic nature typically observed for seed weight in previous soybean studies. The SoyBase database currently contains over 300 quantitative trait loci for seed weight (Karikari et al., 2020), but most represent minor-effect QTLs that collectively explain small proportions of phenotypic variance. Our findings challenge this paradigm by demonstrating that a single major locus can account for a substantial portion of the genetic variation, which has important implications for both breeding strategies and our understanding of seed development biology.

### 4.2 Population genetics and diversity implications

The continuous population structure revealed through principal component analysis, without discrete subpopulations, contrasts with some previous soybean population genetics studies that have identified distinct genetic clusters corresponding to geographic origins or maturity groups (Lam et al., 2010; Zhou et al., 2015). Our findings suggest that extensive germplasm exchange and breeding activities have created a more homogeneous genetic background than previously reported, particularly among elite breeding materials. This continuous structure is advantageous for association mapping as it reduces the risk of spurious associations due to population stratification while maintaining sufficient genetic diversity for QTL detection (Abhijith et al., 2022; Desaint et al., 2023; Altaf et al., 2024). The geographic distribution of our collection, with 81.8% of accessions from China’s primary soybean-producing regions, provides exceptional power for detecting loci relevant to this major production area while maintaining sufficient international representation for broader applicability. Previous GWAS in soybean have often focused on either specific geographic regions or relied heavily on North American breeding materials, potentially limiting the discovery of alleles important in other production environments (Shingote et al., 2022; Zhou and Guo, 2024). Our globally diverse panel bridges this gap by combining extensive sampling from the world’s largest soybean-producing region with representative materials from other major production areas. The consistently higher mean HSW observed in international accessions during 2023 and 2024, coupled with reduced phenotypic ranges compared to Chinese domestic materials, suggests different breeding objectives and selection pressures that align with previous observations about regional breeding programs (Chakelie et al., 2024; Kumari et al., 2025). Historical studies have noted that North American and other international breeding programs have generally focused on larger-seeded varieties for commodity markets, while Chinese breeding has maintained greater diversity, including small-seeded types for food applications. Our quantitative confirmation of these trends provides valuable insights into the global patterns of soybean genetic improvement and their impact on seed size diversity.

### 4.3 Fine-mapping resolution and candidate gene identification

The chr. 20 QTL identified in our study (45.70-46.19 Mb, 493.69 kb interval) overlaps with several previously reported seed weight loci but achieves substantially improved mapping resolution and validation across environments. A comprehensive review of the SoyBase QTL database (Grant et al., 2010), and published literature reveals at least 15 seed weight-related QTLs previously mapped to chr.20, though most were identified through biparental linkage populations with broad confidence intervals, and few have been validated across multiple studies or environments. Previous linkage mapping studies have identified several chr.20 seed weight QTLs with megabase-scale resolution. Luo et al. detected *qSW-20–1* spanning 38–52 Mb (14 Mb interval) in a recombinant inbred line population, explaining 6.8% of phenotypic variance (Luo et al., 2023). Xu et al. mapped *qHSW20* to the 40–48 Mb region (8 Mb interval) with 7.2% variance explained using an F_2_:_3_; population (Xu et al., 2023). Kumar et al. identified a seed shape and weight QTL at 42–49 Mb (7 Mb interval) in a biparental cross (Kumar et al., 2023). While these studies established the importance of chr. 20 for seed weight determination, the broad confidence intervals (7–14 Mb) encompassing hundreds of genes precluded identification of specific candidates and limited immediate breeding applications. Previous GWAS studies have provided evidence for seed weight associations in this chromosomal region with improved resolution compared to linkage mapping, though results have varied in consistency and precision. Zhang et al. conducted GWAS using 309 germplasm accessions and 31,045 SNPs, identifying 22 seed weight loci across the genome but reporting only one minor-effect association on chr. 20 at 30.2-32.8 Mb (2.6 Mb interval, explaining 4.1% variance) a region approximately 13–16 Mb distant from our QTL, suggesting a distinct locus (Zhang et al., 2016). Yan et al. analyzed 166 soybean accessions with the SoySNP50K BeadChip and detected 17 HSW QTLs distributed across six chr. but did not report significant associations on chr. 20, possibly due to limited statistical power from the smaller population size or absence of favorable alleles in their germplasm panel (Yan et al., 2017). More recent and larger-scale GWAS studies have provided converging evidence for seed weight associations overlapping our refined QTL region. Zhao et al. analyzed 809 diverse accessions with 56,110 SNPs and detected a broadly mapped association spanning 43.1-47.3 Mb (4.2 Mb interval) that encompasses our 45.70-46.19 Mb region, explaining 5.9% of variance in a single environment (Zhao et al., 2019). Cao et al. conducted GWAS using 1,024 soybean accessions and identified a major QTL at 44.8-46.5 Mb (1.7 Mb interval, 6.8% variance explained), showing partial overlap with our interval (Cao et al., 2022). Most notably, Karikari et al. employed multiple GWAS models (GLM, MLM, CMLM, SUPER, FarmCPU, and BLINK) with 809 accessions and identified a quantitative trait nucleotide (QTN) at position 45,823,456 bp within *Glyma.20G223400*—remarkably, this is located only 82 kb from our lead SNP at position 45,741,235 bp within *Glyma.20G223200*, providing strong independent validation of this specific genomic region (Karikari et al., 2020).

Resolution improvement and validation advantages of our study compared to previous research include several key advances. First, fine-mapping precision: Our 493.69 kb confidence interval represents a 14-28-fold improvement over traditional linkage mapping (7–14 Mb intervals) and a 3.4-8.5-fold refinement compared to previous GWAS studies (1.7-4.2 Mb intervals), narrowing the region to just 25 candidate genes compared to hundreds in broader intervals. Second, multi-environment stability: Unlike most previous single-environment studies, our QTL maintained genome-wide significance across three consecutive years (2022-2024) with peak -log_10_(P) values of 13.4, 12.1, and 10.2, demonstrating exceptional temporal stability. The consistency across years characterized by different environmental stresses (mean HSW: 20.77 g in 2022, 18.65 g in 2023, 19.41 g in 2024) provides stronger evidence for biological importance than previous reports. Third, larger effect size: Our lead SNP explained 8.7% of phenotypic variance individually and up to 18.7% in multi-SNP models, substantially higher than most previously reported chr.20 QTLs (typically 4-7% variance explained), suggesting either stronger allelic effects in our germplasm or improved statistical power from larger sample size and higher marker density. Fourth, independent validation: The proximity of our lead SNP (45,741,235 bp in *Glyma.20G223200*) to the QTN identified by (Karikari et al., 2020) at 45,823,456 bp provides compelling evidence that this ~80 kb region harbors the causal variant(s) for seed weight variation, as two independent studies with different germplasm panels, genotyping platforms, and statistical methods converged on nearly identical positions.

Our results indicate that the chr.20 region at 45.70-46.19 Mb represents a major, reproducible QTL for soybean seed weight that has been independently detected across multiple studies, populations (biparental crosses, diverse panels, breeding populations), mapping approaches (linkage, GWAS with various models), and geographic regions (China, North America, global collections). Rather than identifying a completely novel locus, our contribution is the substantial refinement of a known major QTL region to a gene-dense interval of 25 candidates with sufficient resolution for positional cloning, combined with rigorous multi-environment validation demonstrating its stability and practical utility for breeding applications. The convergence of evidence from diverse germplasm sources including Chinese landraces, elite breeding lines, North American cultivars, and international accessions indicates this locus harbors broadly relevant genetic variation with consistent effects across genetic backgrounds, making it highly suitable for marker-assisted selection in worldwide soybean improvement programs.

### 4.4 Breeding and applied implications

The identification of a major, stable QTL for HSW has immediate applications for soybean breeding programs. The consistency of this locus across environments suggests that MAS targeting this region could be effective across diverse production systems. The lead SNP, Gm20_45741235, and closely linked markers provide immediate tools for implementing genomic selection strategies, addressing a long-standing limitation noted in previous QTL studies, where identified markers often showed inconsistent effects across environments or populations (Contreras-Soto et al., 2017). The pronounced environmental effects observed across evaluation years (mean reductions of more than 2 g in 2023) underscore the importance of optimizing both genetic potential and environmental management to maximize seed weight. The parallel temporal trends between domestic and international accessions suggest common environmental stresses affecting seed development, emphasizing the need for comprehensive breeding strategies that address both genetic improvement and stress tolerance. The availability of both large-seeded and small-seeded materials within our germplasm collection provides breeding programs with flexibility to develop varieties targeting different market segments, from commodity production to specialty food applications (Li et al., 2015).

## 5 Conclusion

This genome-wide association study using 554 globally diverse soybean accessions identified a major, environmentally stable QTL on Chr. 20 controlling hundred-seed weight. The QTL maintained genome-wide significance across three consecutive years (2022-2024) with peak -log_10_(P) values of 10.2-13.4, explaining 8-12% of phenotypic variance—substantially higher than typical seed weight loci reported previously. Fine-mapping narrowed the critical interval to 493.69 kb containing 25 candidate genes, achieving approximately 10-fold improved resolution compared to traditional linkage-based studies. The lead SNP (Gm20_45741235) within *Glyma.20G223200* provides an immediately actionable molecular marker for breeding applications. Expression analysis revealed up to 32-fold differential expression between contrasting seed size varieties, indicating complex regulatory networks controlling seed development. These findings provide valuable genetic resources for addressing global food security challenges through precision breeding approaches. The robust Chr. 20 QTL establishes a foundation for both continued basic research into seed development mechanisms and immediate application in marker-assisted breeding programs targeting enhanced soybean productivity and seed quality.

## Data Availability

The datasets presented in this study can be found in online repositories. The names of the repository/repositories and accession number(s) can be found in the article/**Supplementary Material**.
